# Loss of Cholinergic and Monoaminergic Afferents in *APPswe/PS1ΔE9* Transgenic Mouse Model of Cerebral Amyloidosis Preferentially Occurs Near Amyloid Plaques

**DOI:** 10.3390/ijms25095004

**Published:** 2024-05-03

**Authors:** Michael K. Lee, Gang Chen

**Affiliations:** 1 Department of Neuroscience, University of Minnesota, Minneapolis, MN 55455, USA; 2 Institute for Translational Neuroscience, University of Minnesota, Minneapolis, MN 55455, USA; 3 Institute for Personalized Medicine in Brain Disorders, The Guangdong-Hongkong-Macao Joint Laboratory of TCM on Brain-Peripheral Homeostasis and Comprehensive Health, School of Chinese Medicine, Jinan University, Guangzhou 510632, China

**Keywords:** Alzheimer’s disease, amyloid, neurodegeneration, cholinergic neurons, monoaminergic neuron

## Abstract

Alzheimer’s disease (AD) is characterized by a loss of neurons in the cortex and subcortical regions. Previously, we showed that the progressive degeneration of subcortical monoaminergic (MAergic) neurons seen in human AD is recapitulated in the *APP_swe_/PS1ΔE9* (APP/PS) transgenic mouse model. Because degeneration of cholinergic (Ach) neurons is also a prominent feature of AD, we examined the integrity of the Ach system in the APP/PS model. The overall density of Ach fibers is reduced in APP/PS1 mice at 12 and 18 months of age but not at 4 months of age. Analysis of basal forebrain Ach neurons shows no loss of Ach neurons in the APP/PS model. Thus, since MAergic systems show overt cell loss at 18 months of age, the Ach system is less vulnerable to neurodegeneration in the APP/PS1 model. We also examined whether the proximity to Aβ deposition affected the degeneration of Ach and 5-HT afferents. We found that the areas closer to the edges of compact Aβ deposits exhibit a more severe loss of afferents than the areas that are more distal to Aβ deposits. Collectively, the results indicate that the APP/PS model recapitulates the degeneration of multiple subcortical neurotransmitter systems, including the Ach system. In addition, the results indicate that Aβ deposits cause global as well as local toxicity to subcortical afferents.

## 1. Introduction

Alzheimer’s disease (AD) is the most common neurodegenerative disease in elderly individuals, characterized by progressive dementia and neurodegeneration. The neurodegeneration in AD is thought to start with the loss of synaptic integrity in the forebrain and the subsequent loss of various neuronal populations, including the cholinergic (Ach) systems in the basal forebrain and monoaminergic (MAergic) systems in the brainstem [1,2,3,4]. The combined findings of current studies indicate that the production of β-amyloid (Aβ) and subsequent formation of senile plaques/Aβ deposits are believed to be the early pathogenic event in AD [5]. However, while numerous in vitro cell culture studies and other studies indicate that aggregated Aβ is neurotoxic [6], a relative lack of neurodegeneration in the mouse models with Aβ pathology supported the view that Aβ pathology is not sufficient to cause progressive neurodegeneration in AD [7,8,9,10,11]. In this regard, we showed that the progressive Aβ pathology in the *APP_swe_/PS1ΔE9* (APP/PS1) Tg mouse model was accompanied by progressive degeneration of the brainstem MAergic systems, including the serotonergic (5-HT) neurons in the raphe and the noradrenergic (NA) neurons in the Locus Coeruleus (LC) [12]. Subsequently, the loss of monoaminergic neurons with amyloid pathology was shown to occur in other mouse models of amyloid pathology [13,14]. Thus, the progressive degeneration of MAergic neurons seen in human AD cases is recapitulated in the APP/PS mouse model, as well as in other mouse models of cerebral amyloid pathology. Importantly, the results suggest that distal toxic factors associated with cerebral Aβ deposits are sufficient to cause progressive degeneration of subcortical MAergic neurons.

In addition to MAergic neurodegeneration, AD is associated with significant loss of the Ach neurotransmitter system, characterized by the loss of forebrain Ach afferents and the selective loss of cholinergic neurons in the subcortical forebrain regions, including the nucleus basalis Meynert (NbM) [1]. Altered Ach function and degeneration contribute to the cognitive decline in AD, while cholinesterase inhibitors, which enhance the Ach function by increasing Ach, are approved for the treatment of AD worldwide [15]. The importance of Ach degeneration to AD’s pathogenesis led to the analysis of the integrity of the Ach system in several Tg models of AD [8,14,16,17]. Some studies show a loss of Ach afferents but no loss of Ach neurons [8,17], while the analysis of the APP^NL-G-F^ and the 5X FAD models shows a significant loss of Ach neurons [14,18]. Although Ach degeneration is very important for the pathology and treatment of AD, the association of Aβ pathology in the distal cortical and hippocampal regions, Ach neurodegeneration, and MAergic neurodegeneration remains unresolved.

Here, using unbiased stereological methods, we extended our previous study on the MAergic neurotransmitter system in the APP/PS mouse model by examining the integrity of the Ach system in the APP/PS mouse model. Further, while studies suggest that Aβ deposits exert a toxic effect on the nearby neuronal processes and terminals, such as increased axonal dystrophy, loss of synaptic markers, and neuronal loss [19,20,21], it is unknown if the degeneration of afferents from subcortical neurons is influenced by their physical distances from Aβ plaques. To address this issue, we assessed the spatial relationship between the distance from Aβ depositions and Ach or 5-HT neurodegeneration. We found that a progressive cortical Aβ pathology is associated with degeneration of Ach afferents in the APP/PS1 model and a modest loss of NbM Ach neurons at older ages. Moreover, we demonstrate that with the development of Aβ pathology, greater reductions in the densities of Ach and 5-HT fibers occur near the Aβ plaques than in the areas that are distal to the Aβ plaques. 

## 2. Results

### 2.1. The Progressive Loss of Ach Afferents in the Cortex and Hippocampus follows the Loss of MAergic Afferents

**Figure 1:**
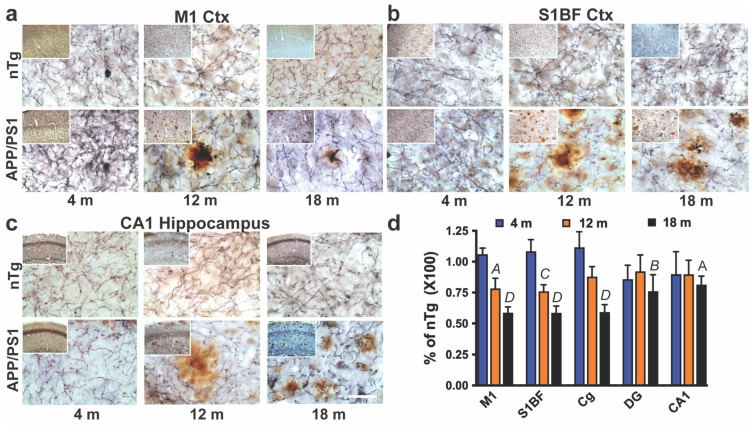
Progressive degeneration of Ach axons in the cortex and hippocampus of APP/PS1 model. (**a**–**c**). Representative micrographs of Ach fibers (dark blue) stained with anti-ChAT antibody and amyloid plaques (brown) stained with 6E10 antibody in the Primary motor cortex (M1 Ctx, (**a**)), Barrel Field (S1BF, (**b**)), and CA1-hippocampus (**c**) area at 4-, 12-, and 18 months (m) of age. Inset shows a lower magnification image showing an increased number of amyloids in older mice. Scale bar = 50 µm, 250 µm inset. Larger images of panels a-c are presented in Figure S1. (**d**). Ach fiber density in APP/PS1 mice, relative to nTg mice, in the Primary motor cortex (M1), Primary Sensory Barrel Field (S1BF), cingulate cortex (Cg), dentate gyrus–hippocampus (DG), and CA1 in 4-, 12-, and 16-month-old mice. Mean ± SEM, significance values are comparison to nTg shown in Figure S2. *A*, *p* < 0.05; *B*, *p* < 0.01; *C*, *p* < 0.001; *D*, *p* < 0.0001 vs. nTg, ANOVA with Tuckey’s post-test (see Figure S2), n = 6 for 4 m and 12 m subjects, n = 7 for 18 m subjects.

To determine whether a progressive Aβ pathology in the APP/PS1 Tg mouse model is associated with the progressive loss of Ach neurotransmitter systems, we used the brain sections from animals previously used for the analysis of MAergic neurotransmitter systems [12]. Previous analysis of these animals showed that progressive Aβ deposition was associated with a ~50% and ~70% loss of forebrain MAergic afferents at 12 months and 18 month of age, respectively [12]. Thus, the use of these sections will allow us to directly compare the spatial and temporal pattern of Ach afferent integrity in relation to the changes in the MAergic afferents in the same animals. The serial sections from 4-, 12-, and 18-month-old mice were first stained for ChAT to visualize Ach afferents, followed by 6E10 immunostaining to visualize Aβ deposits. We sampled the Primary Sensory Barrel Field (S1BF), primary motor (M1), cingulate cortices (Cg), and hippocampal regions (CA1 and dentate gyrus, DG) from the stained sections to determine the integrity of ChAT+ afferents (Figure 1a, Figures S1 and S2). The overall length/density of the ChAT+ afferents was determined stereologically using spherical probe analysis [12,22]. At 4 months of age, there were very few amyloid deposits in the APP/PS1 mice, and the overall integrity of the Ach afferents in different brain regions of APP/PS1 mice was comparable to that seen in the littermate nTg mice. With the increase in the Aβ deposition in 12-month-old APP/PS1 mice, a quantitative analysis of the Ach afferents showed that the density of Ach afferents in M1, S1BF, and Cg was trending lower (~20–25% reduction) than in the nTg mice. However, the differences between the 12-month-old nTg and APP/PS1 mice did not achieve statistical significance (Figure 1b). With the further progression of amyloid deposits at 18 months of age, the Ach afferent densities in the forebrain brain regions of the APP/PS1 mice were significantly lower (~35–45%) than in the mice from all other genotypes (Figure 1b). Singly, Tg mice (APP-alone, PS1-alone) were not different from each other or nTg mice at 12 and 18 months of age, confirming that the loss of Ach afferents, as with the MAergic afferents, is only associated with progressive Aβ pathology (Figure S2). The time course of Ach afferent loss is significantly slower than with the MAergic afferents, where the loss is ~50% at 12 months of age and 75–80% at 18 months of age. Moreover, with the MAergic afferents, there was a significant loss in the hippocampus of APP/PS1 mice [12]. 

### 2.2. Modest Loss of Ach Neurons in the Basal Forebrain Occurs with Aging in the APP/PS1 Model

**Figure 2:**
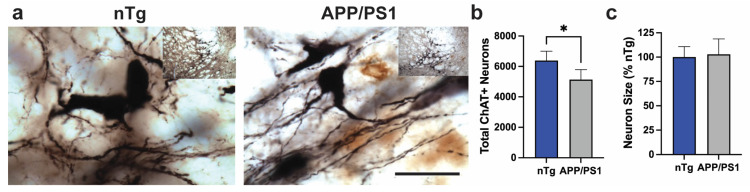
A modest loss of Ach neurons in NbM without neuronal atrophy. (**a**). Representative micrographs of ChAT immunoreactive neurons in the Nucleus Basalis of Meynert (NbM) of 18-month-old nTg and APP/PS1 mice. Lower-magnification images are inserted in the left corners of images with higher magnifications. Scale bar = 50 µm, 250 µm inset. (**b**). Stereological analysis of total ChAT+ neurons in the NbM shows a modest but significant loss of neurons in APP/PS1 mice. Mean ± SEM, * p < 0.05, n = 4 per group. (**c**). Stereological analysis of average neuron size shows no difference between nTg and APP/PS1 mice. n = 4 per group.

To determine whether the moderate loss of Ach afferents is reflected as a loss of neurons or neuronal shrinkage at 16 months of age, the size and number of Ach neurons in the basal forebrain (NbM) were determined from ChAT immunostained sections (Figure 2). The analysis shows that a moderate reduction in cortical Ach afferents at 18 months of age was associated with a modest (~20%) but significant loss of ChAT-positive neurons in the NbM (Figure 2c). However, there was no change in the relative size of the Ach neurons in the NbM (Figure 2c). The result is consistent with other studies showing moderate to modest loss of Ach neurons in the mouse model of cerebral amyloid pathology [8,17]. Overall, our results show that Aβ pathology in the APP/PS1 Tg mouse model is associated with the progressive degeneration of both MAergic and Ach neurotransmitter systems, both of which are present in AD cases [1,23]. However, the Ach pathology is less severe than the MAergic pathology, as these mice exhibit >70% loss of MAergic afferents and ~50% loss of MAergic neurons at 18 months of age [12], which is consistent with the degeneration pattern in human AD cases [23]. 

### 2.3. Spatial Relationship between Degeneration of Ach and 5-HT Afferents and Aβ Pathology

Previous studies showed that Aβ deposits may exert local toxicity that decreases with distance from the deposits [19,24]. Thus, we examined whether the losses of MAergic and Ach afferents were differentially affected as a function of distance from Aβ deposits. To determine if the integrity of subcortical afferents is a function of distance from Aβ deposits, brain sections were stained for ChAT or Serotonin (5-HT) (dark blue/black), followed by immunostaining for Aβ deposits using 6E10 antibody (brown). 

**Figure 3:**
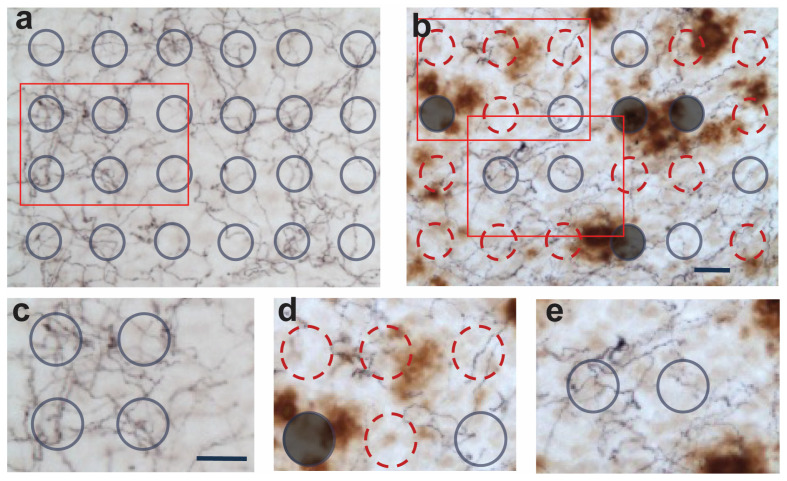
Schematic for analyzing the integrity of afferents as a function of distance from Aβ deposits. (**a**,**b**). Representative micrographs of cortex from 12-month-old nTg (**a**) and APP/PS1 (**b**) stained for 5-HT (dark blue/black) afferents, followed by Aβ (6E10, brown). Shown are examples of counting frames laid out by the stereology system. The grey circles are distant from Aβ, red circles are near Aβ, and filled circles are within Aβ and excluded. (**c**–**e**). A higher magnification view of red inset areas from nTg (**c**) and APP/PS1 (**d**,**e**) mice. Bar = 20 μm.

For the length estimation with spherical probes, each probe (10 μm diameter) was marked as distant, near, or within an Aβ deposit based on the distance of the probe’s radial center from the edge of the Aβ deposit (≤20 μm—near, >20 μm—distant). Probes whose radial center fell within the edge of Aβ deposits were considered “within” and were not considered (Figure 3). 

**Figure 4:**
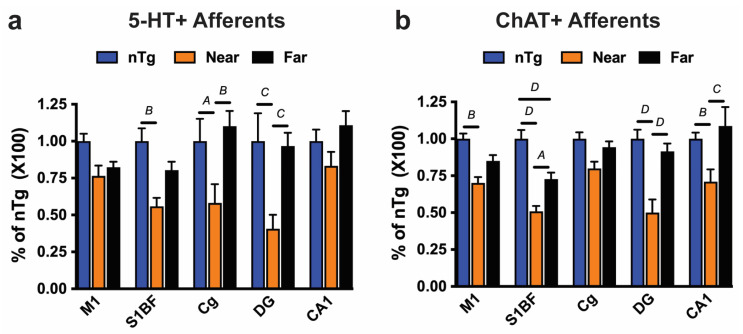
Aβ deposit exerts local toxicity. Using the scheme outlined in Figure 3, the density of 5-HT+ (**a**) and ChAT+ (**b**) afferents near or far from Aβ deposits were determined in 12-month-old mice. The afferent densities were normalized to the afferent density seen in disease-free nTg mice and plotted as mean ± SEM. In all cases, the overall densities of the afferents near the amyloid deposits are lower than either nTg or far from amyloid deposits. *A*, *p <* 0.05; *B*, *p <* 0.01; *C*, *p <* 0.001; *D*, *p* < 0.0001; two-way ANOVA, Tukey’s multiple comparison test, n = 6 animals per group.

Because we previously showed that MAergic afferents are particularly vulnerable to the toxic effects of Aβ pathology, we first compared 5-HT afferent density in areas near to (≤20 μm) or distant (>20 μm) from the Aβ deposits (Figure 4a) in 12-month-old APP/PS1 Tg mice in whom pronounced MAergic neurodegeneration was already in place [12]. As a reference for the normal density of 5-HT afferents, we used the values from the nTg mouse. Different brain regions exhibited different levels of reduction in 5-HT fiber density near the Aβ deposits, compared to those that were distant from the deposits: there was a severe reduction (about 60%) in the hippocampal DG, medium (about 45%) in the Cg, and mild (about 20–30%) in the S1BF and CA1. Significantly, the 5-HT afferent density in areas that were distant from Aβ deposits was not significantly different from that seen in nTg mice. This result indicates that Aβ deposits exert local toxicity that is reduced with distance from the Aβ deposit. 

We also examined whether the Ach afferents are differentially affected as a function of distance from Aβ deposits. Using the spatial scheme used for 5-HT afferents, we examined the integrity of Ach afferents near or distant from Aβ deposits. The analysis of the 12-month-old mice showed that the Ach afferent density near the Aβ deposits was significantly lower than the afferent densities in both the areas distant from Aβ deposits and the reference density in the nTg mice (Figure 4b). Further, except for S1BF, there was no significant difference in ChAT+ afferent densities between areas that were distant from Aβ deposits and the nTg reference. While the overall ChAT+ afferent densities in different brain regions of 12-month-old APP/PS1 mice were not significantly different from nTg mice (Figure 1d), our analysis revealed that the ChAT+ afferents were significantly affected by Aβ pathology at 12 months of age. Moreover, the amyloid-associated reduction in the Ach afferent densities seen at 12 months of age (Figure 1d) was almost entirely due to the loss of afferents near the Aβ deposits. It is interesting to note that while the ChAT+ afferent was severely affected by Aβ deposits in the hippocampus (DG, CA1), the overall density of ChAT+ afferents was not different from that of nTg mice, because the hippocampus exhibits less Aβ pathology at 12 months of age (Figure S4). As expected, the loss of ChAT+ afferent densities near the Aβ deposits was more severe at 18 months of age than at 12 months of age (Figure S4). The progressive nature of neurodegeneration is also indicated by the significant loss of ChAT+ afferents in areas that were distant from Aβ deposits at the S1BF, M1, and Cg of 18-month-old APP/PS1 mice (Figure S4). 

## 3. Discussion

The progressive degeneration of the Ach neurotransmitter system is one of the most constant neurodegenerative features of AD [15,16] and has been widely accepted as crucial for the cognitive decline in AD [15,16]. We used the APP/PS1 Tg model of AD, which has been previously shown to recapitulate progressive MAergic degeneration in AD [12], to determine the relation between Aβ pathology and the integrity of the Ach neurotransmitter system. We found that a severe Aβ pathology is associated with the progressive degeneration of Ach axons, where the global loss of cortical Ach afferents is apparent by 18 months of age. Thus, the loss of Ach afferents occurs later than the loss of MAergic afferents in this model, where significant global loss is seen by 12 months of age [12]. Further, consistent with the relatively moderate loss of Ach afferents in 18-month-old APP/PS1 mice, there was only a modest but significant loss of Ach neurons, without obvious neuronal atrophy. Our previous study showed that at the same age, almost half of the MAergic neurons are lost in Raphe and Locus Coeruleus [12]. Because studies suggest that the Aβ deposit may exert toxic effects within the local vicinity of the deposits [19,20], we also investigated the spatial relationship between Aβ deposition and the loss of 5-HT+ and ChAT+ axons in selective cortical and hippocampal regions. We found that areas near the Aβ deposits showed a lower density of Ach and 5-HT axons than the areas that were more than 20 μm away from the edge of the Aβ deposits. These findings suggest that progressive Aβ depositions in the cortex and hippocampus with aging in the APP/PS1 mice may have a role in Ach and MAergic axonal degeneration. 

Because Ach degeneration is directly linked to cognitive dysfunction in AD, the integrity of the Ach system has been studied in several Tg models of AD [8,14,16,17]. In general, our studies are in agreement with these prior studies, where axonal loss occurred with advanced aging (16+ months old), with no loss of Ach neurons [8,17], including the analysis of the same APP/PS1 mouse lines analyzed here. In this regard, both the APP^NL-G-F^ mutant mice and the 5X FAD model seem to exhibit a significant loss of Ach neurons as early as 6 months of age [14,18]. Thus, the current and prior studies support the view that Aβ pathology is sufficient to cause progressive neurodegeneration. It is clearly interesting to note that both Ach (this paper) and MAergic afferents [12] in S1BF seem to exhibit increased vulnerability to Aβ pathology. For example, at 12 months of age, only S1BF exhibited a significant loss of ChAT+ afferent fibers. Previous studies in the same Tg model have also demonstrated S1BF, with the most remarkable degeneration of MAergic fibers. One reason for this might be that Aβ pathology is more severe in S1BF, particularly compared to the hippocampus (Figure S3).

**Figure 5:**
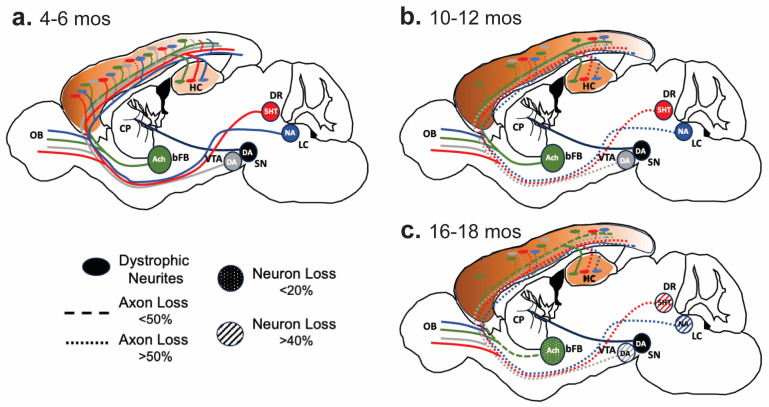
The Pattern of progressive degeneration of the subcortical neurotransmitter system in an APP/PS1 model. (**a**). At 4–6 months of age, when Aβ pathology (Brown) starts to appear, MAergic (5-HT, Red; NA, Blue; DA-SNpc, Black, DA-VTA, Grey) and Ach (Green) afferents show dystrophic terminals around the Aβ deposits. (**b**). At 10–12 months of age, with the increase in Aβ pathology, significant loss (~40–50%) of MAergic afferents is seen, along with atrophy of MAergic cell bodies [12]. In contrast, while local losses of ChAT+ afferents around the Aβ deposits occur, there is no significant global loss of Ach (ChAT+) afferents. (**c**). In a 16–18-month-old APP/PS1 mouse model, a continued loss of MAergic afferents leads to an overt loss of MAergic neurons. The losses of ChAT+ afferents are now significant (~40%) in certain areas, with modest neuronal loss (<20%). 5-HT, Serotonin; Ach, Acetylcholine; bFB, basal forebrain; CP, Caudate Putamen; DA, Dopamine; DR, Dorsal Raphe; HC, hippocampus; LC, Locus Coeruleus; NA, noradrenaline; OB, Olfactory Bulb; SN, Substantia Nigra; VTA, Ventral Tegmental Area.

The studies on neurodegeneration in the APP/PS1 mouse model suggest that progressive neurodegeneration in the APP/PS1 model occurs via a dying-back process, where axon terminals and axonal degeneration occur first, followed by the loss of neurons. The dying-back neurodegeneration is likely to occur in AD, as this pattern of neurodegeneration is consistent with the more severe loss of axons in the earlier stages of AD [25]. Based on the patterns of neurodegeneration in the APP/PS1 mouse model, we also propose that the vulnerability of subcortical neurons to the neurotoxic effects of Aβ pathology is related to the length of afferents into forebrain areas that develop Aβ pathology (Figure 5). Based on this hypothesis, we would expect that in the APP/PS1 model, subcortical MAergic neurotransmitter systems would be more vulnerable to progressive neurodegeneration than the basal forebrain Ach neurons. Moreover, mouse models of cerebral Aβ pathology exhibit only modest neurodegeneration in the cortex and hippocampus and only at rather advanced ages [8,9,26], presumably because cortical and hippocampal neurons have rather short neuritic (axons and dendrites) connections to areas with Aβ pathology. We propose that the neuronal vulnerability related to the length of projections to areas with Aβ pathology might be why humans, having larger brains with longer axons, exhibit more global neurodegeneration. 

Although recent studies seem to have moved away from compact amyloid toxicity to soluble Aβ oligomer as a toxic agent, there is still ample evidence suggesting that the Aβ deposits have local toxic effects [19,20,21]. Here, we found that using systematic unbiased sampling approaches, ChAT+ or 5-HT+ axons close to the plaques exhibited a greater degree of degeneration than those that were remote to the plaques, suggesting that the Aβ plaques or Aβ plaque-associated components may contribute to the gradual loss of axons originating from the subcortical regions. In agreement with neuropathological findings from our studies and others, it was reported that neurons or dendritic segments that were close to plaques exhibited diminished neuronal activity compared with those that were remote to plaques in the Tg mice, indicating that Aβ deposits seen in the Tg mouse models are not benign, as they are a focal lesion leading to impaired neural system function. 

## 4. Materials and Methods

### 4.1. Subjects

The tissues used in these studies were from the same subjects previously analyzed for the integrity of MAergic neurons [12]. The mice comprised all possible genotypes, generated by mating MoPrp-Mo/Hu *APP_swe_* (line 3-3) with *MoPrp-PS1ΔE9* (line S9) Tg mice [12]. Because we analyzed tissue sections from a previous study, no live animals were used in this study. We used animals at 4-, 12-, and 18 months of age. These ages were chosen because 4-month-old mice are at the initial stages of amyloid pathology; 12 months represents a stage where the mice show episodic memory deficits and ~50% loss of MAergic afferents [12,27]; and 18 months represent a stage when the mice exhibit reference memory deficits and overt loss of MAergic cell bodies [12,27]. 

### 4.2. Histology

For histological analysis, the tissues used were fixed with 4% paraformaldehyde, and serially frozen coronal brain sections (40 μm) were obtained as described [12]. To detect Ach or 5-HT fibers and neurons and amyloid deposits in the same brain section, a serial double-immunocytochemical staining procedure was employed. Free-floating frozen sections were first immunodetected with an anti-ChAT antiserum (AB143, MilliporeSigma, Burlington, MA, USA) or anti-5-HT antiserum (Immunostar, Hudson, WI, USA). Sections were then incubated with the appropriate 2nd antibody, followed by the ABC method (Vector Laboratories, Newark, CA, USA), using diaminobenzidine–nickel (DAB-Ni) as the chromogen to obtain the dark blue/black reaction products. The sections were then immunoreacted with anti-Aβ mouse monoclonal antibodies (6E10, Covance, SIG-39300, Burlington, NC, USA), and DAB was used as the chromogen to obtain brown color products. We also used another anti-Aβ mouse monoclonal antibody (4G8, Covance, SIG-39200, Burlington, NC, USA) for the detection of amyloid pathology.

### 4.3. Stereological Analysis of Afferent Density

The stereological length estimation with spherical probes (Stereo Investigator; MicroBrightField, Williston, FL, USA) was used to determine the length of Ach fibers [28], with a modification used for the analysis of MAergic afferents [12]. To account for potential regional and rostrocaudal heterogeneity of Ach afferents, we focused our analysis on the selected subregions defined by *The Mouse Brain in Sterotaxic Coordinates* [29] and analyzed every twelfth section spanning the region of interest, as previously defined [12]. In general, this sampling led to analyzing 3–4 sections per subject. Virtual spherical probes, concentric circles of progressively increasing and decreasing diameters, were placed within a 40 μm thick section to determine the axon length. At each focal plane, the intersections between the immunoreactive fibers and circles were counted (*Q*) under 100× objective. This method allows for the simple determination of the total length density (*L_V_*) and the total length (*L*) [28]. To reduce the effects of variations in the area selection, *L_V_* was routinely used for comparison between groups. The axon lengths were measured at 50 random locations through the reference space. 

To assess whether the Aβ deposits have differential effects on the degeneration of Ach or 5-HT fibers, each spherical probe was marked as within, near, or distant from an Aβ deposit. A probe whose radical center was less or larger than 10 μm from the periphery of a deposit was defined as the proximal or distant probe, respectively (Figure 3). A probe whose radical center fell within the edge of Aβ deposit was defined as a probe “within” the deposits and was not factored into our analysis. In our analysis, we could not determine the actual length of the fibers, but the number of fibers crossing per probe was used as a surrogate for the length estimation. Because fibers crossing per probe are directly proportional to the actual length, the differences in the values reflect the actual fiber densities near and distant from the Aβ deposits. 

### 4.4. Stereological Analysis of Neuron Number/Size

The total ChAT+ neuron numbers were estimated using the optical fractionator (StereoInvestigator; MicroBrightField) [12,22] by analyzing every sixth section through the entire region containing the Basal nucleus of Meynert. In general, we used a 100 × 100 μm counting frame, a 1 μm guard, a 200 × 200 μm sampling grid, and a dissector height of 10 μm. Unbiased stereological analysis of neuronal size (area and volume) were performed using a vertical nucleator probe, as previously described [12,22]. 

### 4.5. Statistical Analysis

All statistical analyses were performed using the Prism software (Prims 10, GraphPad, San Diego, CA, USA). The statistical analysis included the two-tailed *t* test and one- or two-way ANOVA. When F values showed significance at a level of *p* < 0.05, Fisher’s post hoc analysis or Newman–Keuls post-test was applied to determine where the differences among groups arose. Data represent mean ± SEM for each group of animals. 

## Data Availability

The datasets and images used and/or analyzed for the current study are available from the corresponding author on reasonable request.

## Supplementary Materials

The following supporting information can be downloaded at https://www.mdpi.com/article/10.3390/ijms25095004/s1.
