# Supplementary material for: Loss of Cholinergic and Monoaminergic Afferents in APPswe/PS1ΔE9 Transgenic Mouse Model of Cerebral Amyloidosis Preferentially Occurs Near Amyloid Plaques
Source: Int J Mol Sci. 2024 May 3;25(9):5004. doi: 10.3390/ijms25095004 (PMC11084680; doi:10.3390/ijms25095004)
Supplement: Supplementary file 1 [file ijms-25-05004-s001.zip › ijms-2963308-supplementary.pdf]

Supplementary File 1

Supplementary Figures S1-S4

For

Loss of cholinergic and monoaminergic afferents in transgenic mouse model of cerebral amyloidosis preferentially occur near the amyloid Plaques.

Gang Chen and Michael K. Lee

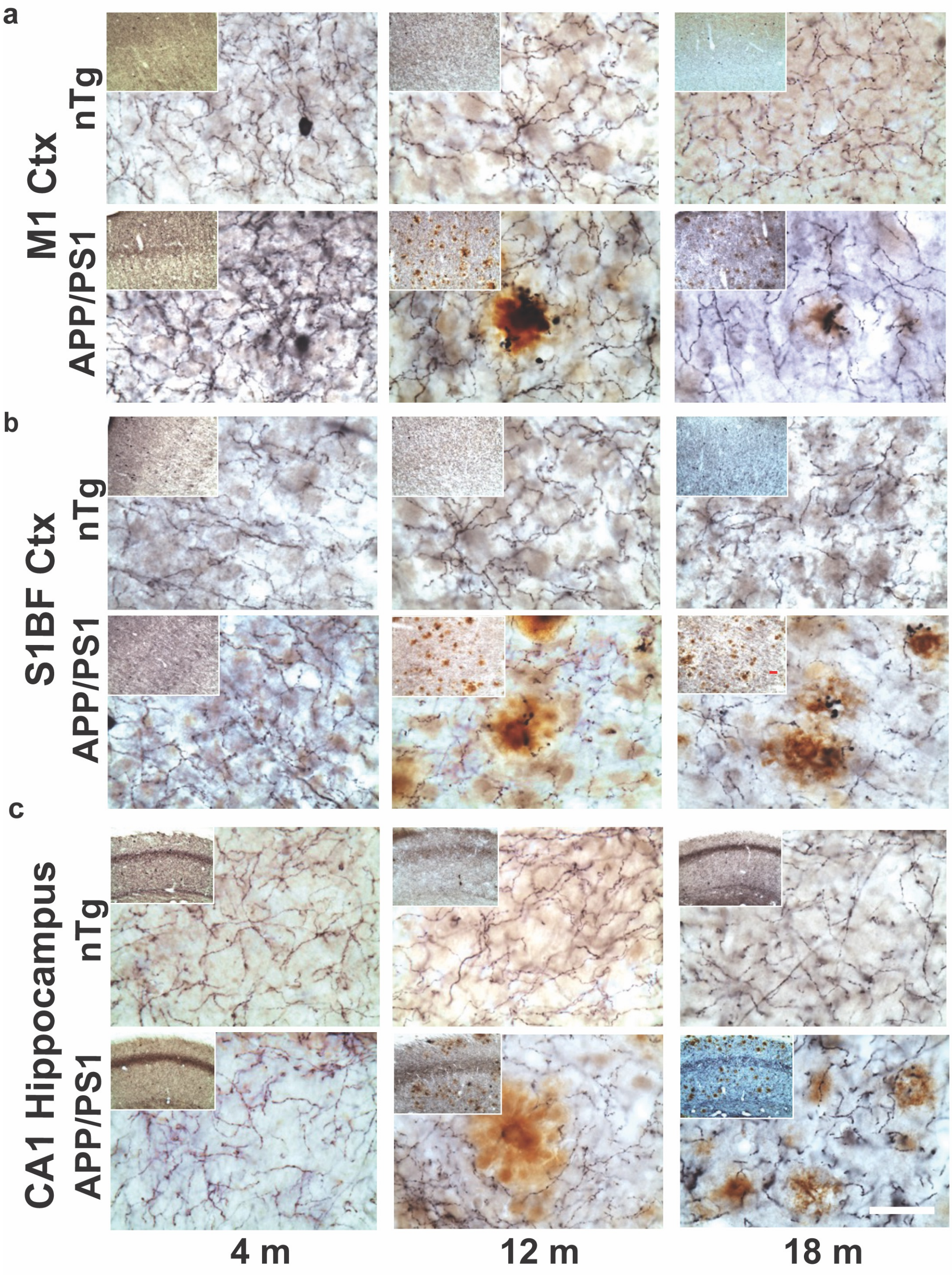

Figure S1. Large images showing Ach axons in the cortex and hippocampus of APP/PS1 model. Representative micrographs of Ach fibers (dark blue) stained with anti-ChAT antibody and amyloid plaques (brown) stained with 6E10 antibody in the Primary motor cortex (M1 Ctx, **a)**, Barrel Field (S1BF, **b)**, and CA1-Hippocampus **(c)** area at 4-, 12-, and 18-months (m) of age. Inset show a lower magnification image showing increased number of amyloid deposits in older mice. Scale bar=50 μm,

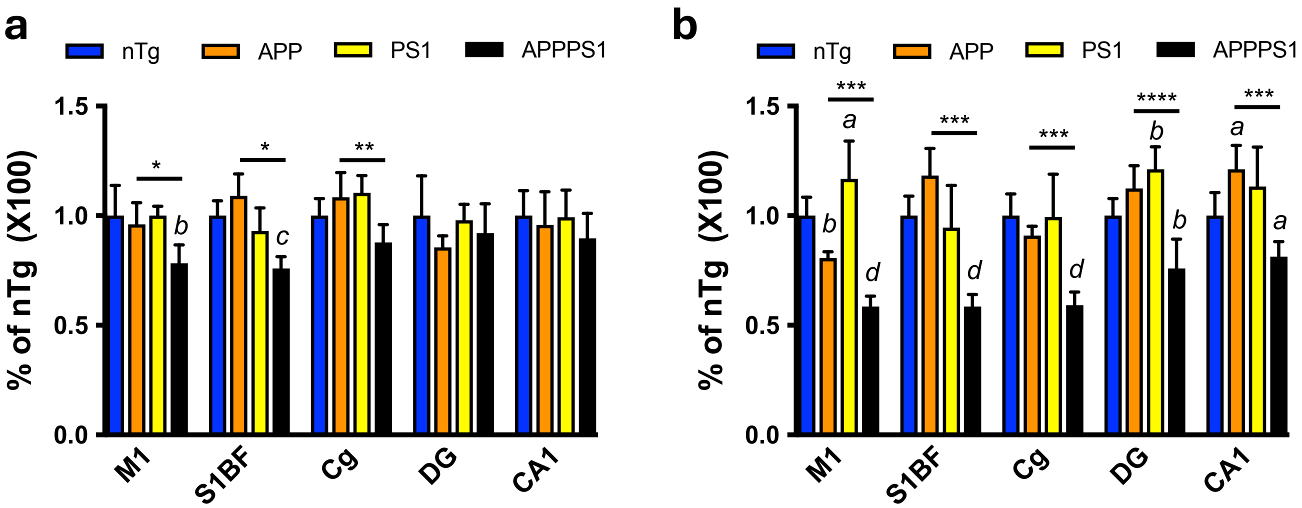

Figure S2. ChAT+ fiber density, relative to the average of nTg mice, in all genotypes at 12 months **(a)** and 18 months **(b)** of age. Plotted are mean ± SD. **a)** Significantly lower ChAT+ afferents, compared to nTg, are seen in cortical regions (M1, S1BF) from APP/PS1 mice. *b*, *p<0.01; c, p<0.001* vs nTg. One-way ANOVA, Tukey's multiple comparison test, *n=6*. There is no differences in ChAT+ afferent integrity in Cg, DG, or CA1 compared to nTg mice. However, ChAT+ afferent density is lower in Cg APP/PS1 mice compared to the APP-alone or PS1-alone. **p<0.05, **p<0.01*, APP/PS1 vs APP-alone/PS1-alone, One-way ANOVA, Tukey's multiple comparison test. **b)** APP/PS1 mice show significant loss of ChAT+ afferents at 18 months of age in all brain regions. Single transgenic APP and PS1 mice are comparable to nTg mice except where indicated. *a, p<0.05, b, p<0.01; d, p<0.0001* vs nTg. One-way ANOVA, Tukey's multiple comparison test, *n=7. ***p<0.001, ****p<0.0001,* APP/PS1 vs APP-alone/PS1-alone, One-way ANOVA, Tukey's multiple comparison test. Primary Motor Cortex (M1), Primary Sensory Barrel Field (S1BF), Cingulate Cortex (Cg), Dentate gyrus-Hippocampus (DG), and CA1-hippocampus.

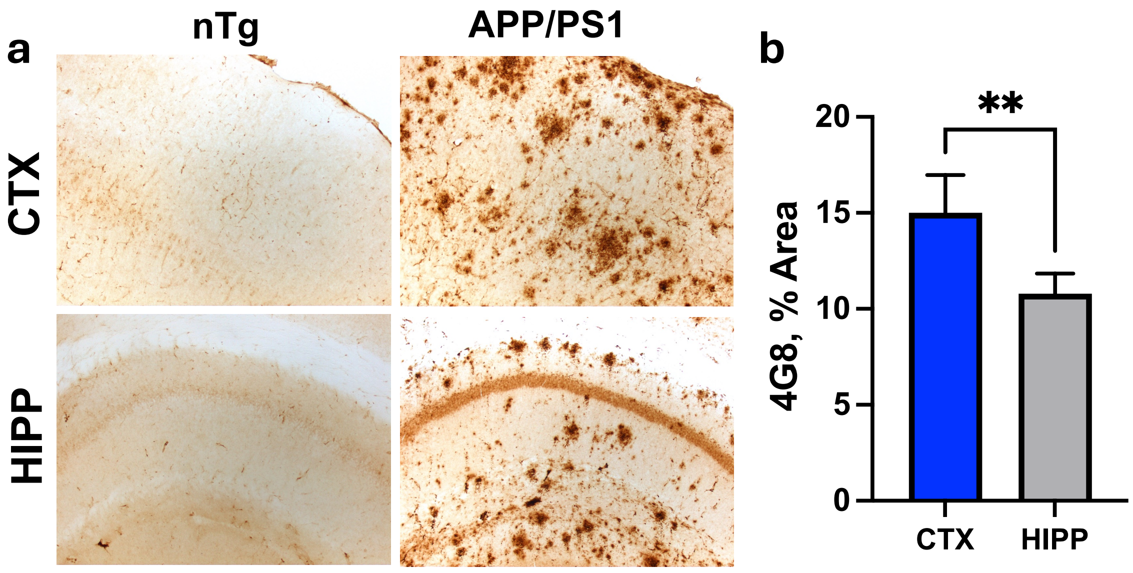

Figure S3. Amyloid pathology is higher in the cortex than in the hippocampus. **a)** Representative 4G8 immunostained images of Cortex (CTX) and CA1 areas of Hippocampus (HIPP) of 12-month-old nTg and APP/PS1 mice. **b)** Quantitative analysis of the total area covered by 4G8 immunoreactivity (mean ± SEM) showing higher levels of amyloid pathology in CTX. *$**p<0.01$, unpaired t-test*, n=4.

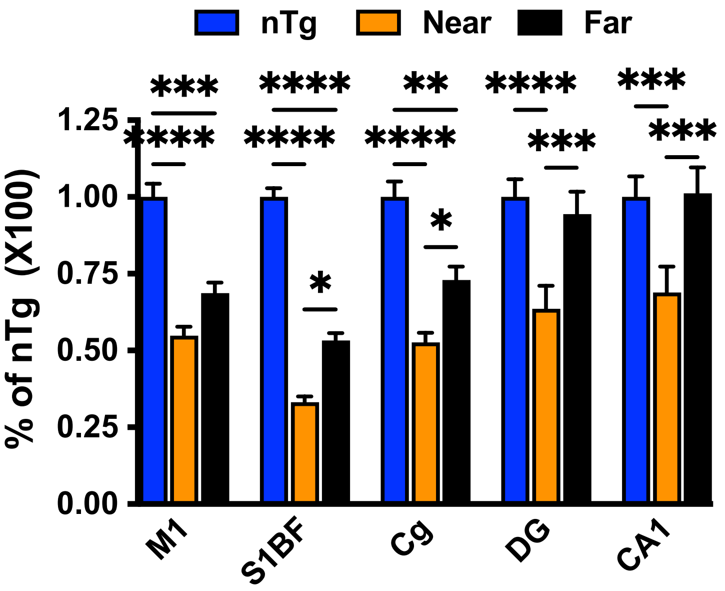

Figure S4. Using the scheme outlined in Figure 3, ChAT+ fiber density in 18-month APP/PS1 animals w as determined as a function of distance from Aβ deposits. The values were normalized to average density in nTg mice. Unlike in 12-month-old mice, some brain areas (M1, S1BF, and DG) show global loss of ChAT+ afferents at both near and far from Aβ deposits. Plotted are mean ± SEM. **p<0.05, **p<0.01, ***p<0.001, ****p<0.0001,* Two-way ANOVA, Tukey's multiple comparison test, n=7.
